# Rates and Trends of Pediatric Acute Lymphoblastic Leukemia — United States, 2001–2014

**DOI:** 10.15585/mmwr.mm6636a3

**Published:** 2017-09-15

**Authors:** David A. Siegel, S. Jane Henley, Jun Li, Lori A. Pollack, Elizabeth A. Van Dyne, Arica White

**Affiliations:** ^1^Epidemic Intelligence Service, CDC; ^2^Division of Cancer Prevention and Control, National Center for Chronic Disease Prevention and Health Promotion, CDC.

Acute lymphoblastic leukemia (ALL) is the most prevalent cancer among children and adolescents in the United States, representing 20% of all cancers diagnosed in persons aged <20 years, or >3,000 new cases each year (*1*). Past studies reported increasing trends of ALL overall and among Hispanics, but these represented ≤28% of the U.S. population and did not provide state-based estimates (*1*–*3*). To describe U.S. ALL incidence rates and trends among persons aged <20 years during 2001–2014, CDC analyzed rigorous data (based on established publication criteria) from the United States Cancer Statistics data set, which includes incidence data on approximately 15,000 new cases per year of all types of invasive cancer among children and adolescents aged <20 years (*4*). The data set represented 98% of the U.S. population during the study period. Overall incidence of pediatric ALL during 2001–2014 was 34.0 cases per 1 million persons and among all racial/ethnic groups was highest among Hispanics (42.9 per 1 million). Both overall and among Hispanics, pediatric ALL incidence increased during 2001–2008 and remained stable during 2008–2014. ALL incidence was higher in the West than in any other U.S. Census region. State-specific data indicated that the highest rates of pediatric ALL incidence were in California, New Mexico, and Vermont. These demographic and geographic ALL incidence data might better inform public health interventions targeting the following areas: exposures to recognized risk factors for leukemia; ALL treatment, including clinical trial enrollment; survivorship care planning; and studies designed to understand the factors affecting changes in pediatric cancer incidence.

The United States Cancer Statistics data set includes cancer incidence data from CDC’s National Program of Cancer Registries and the National Cancer Institute’s Surveillance, Epidemiology, and End Results program (*4*). Data on new cases of cancer diagnosed during 2001–2014 were obtained from population-based cancer registries affiliated with the National Program of Cancer Registries or Surveillance, Epidemiology, and End Results programs. Incidence data for all registries except the District of Colombia, Mississippi, and Nevada met United States Cancer Statistics publication criteria during 2001–2014, and represented 98% of the U.S population.* This report includes cases diagnosed among children and adolescents aged <20 years and includes *International Classification of Diseases for Oncology, Third Edition*^†^ codes 9728–9729, 9811–9818, and 9835–9837 as grouped by the *International Classification of Childhood Cancer*.^§^ Cases were included if ALL was the first or only cancer diagnosed and was confirmed microscopically or by positive laboratory test or marker study. Recurrent cases of ALL were not included in this report.

Age-adjusted rates were calculated using statistical software. All rates were expressed per 1 million persons and were age-adjusted to the 2000 U.S. standard population.^¶^ Age-adjusted incidence trends were quantified using annual percent change (APC) calculated using joinpoint regression. Statistically significant APCs were different from zero (p<0.05). A maximum of two joinpoints were used to determine a change of direction in trends during the study period. Rates and trends were estimated by sex, age group, race/ethnicity, state, U.S. Census region,** county-level economic status, and rural/urban status.

During 2001–2014, a total of 38,136 new pediatric ALL cases were diagnosed in the United States (Table). Overall incidence of ALL was 34.0 cases per 1 million. Rates were highest in children aged 1–4 years (75.2 per 1 million) and were higher in males (38.0) than in females (29.7). Among all racial/ethnic groups, the highest incidence rate (42.9 per 1 million) was among Hispanics, followed by non-Hispanic whites (34.2 per 1 million). The lowest incidence (18.7) occurred among non-Hispanic blacks. Pediatric ALL incidence rates in the 25% of U.S. counties with the highest economic status were higher than rates in the 25% of counties with the lowest economic status and were higher in metropolitan areas with ≥1 million persons than in nonmetropolitan areas. Rates were highest in the West (38.5) followed by the Northeast (34.8), Midwest (32.4), and South (31.6) Census regions, and, among states, were highest in Vermont (41.9), California (40.8), and New Mexico (39.1) (Figure 1) (Supplementary Table 1, https://stacks.cdc.gov/view/cdc/47662). State-specific ALL incidence by race/ethnicity ranged from 10.1–27.9 per 1 million among non-Hispanic blacks to 25.1–45.0 among non-Hispanic whites and 27.3–48.5 among Hispanics (Supplementary Table 2, https://stacks.cdc.gov/view/cdc/47663).

**TABLE:** Age-adjusted incidence* of acute lymphoblastic leukemia^†^ in persons aged <20 years and annual percentage change (APC) in rates, by selected characteristics — United States,^§^ 2001–2014

Characteristic	No.	Incidence (95% CI)	APC^¶^					
			Years	APC_1_ (95% CI)	Years	APC_2_ (95% CI)	Years	APC_3_ (95% CI)
**Overall**	**38,136**	**34.0 (33.6–34.3)**	**2001–2008**	1.9 (0.5–3.3)**	**2008–2014**	**-1.1 (**-**2.8–0.6)**	**—^††^**	**—**
**Sex**								
Male	21,871	38.0 (37.5–38.5)	2001–2008	2.1 (0.5–3.7)**	2008–2014	-1.5 (-3.3–0.4)	—	—
Female	16,265	29.7 (29.2–30.1)	2001–2003	-4.0 (-14.7–8.1)	2003–2008	3.2 (-0.5–7.0)	2008–2014	-1.0 (-2.9–0.9)
**Age group (yrs)**								
<1	1,009	18.4 (17.3–19.6)	2001–2014	-1.5 (-3.3–0.3)	—^††^	—	—	—
1–4	16,388	75.2 (74.0–76.4)	2001–2009	1.3 (-0.1–2.8)	2009–2014	-2.4 (-5.2–0.5)	—	—
5–9	9,535	34.8 (34.1–35.5)	2001–2010	2.2 (1.3–3.2)**	2010–2014	-1.7 (-4.6–1.3)	—	—
10–14	6,201	21.6 (21.1–22.1)	2001–2014	1.3 (0.5–2.1)**	—	—	—	—
15–19	5,003	17.0 (16.5–17.5)	2001–2014	0.4 (-0.5–1.3)	—	—	—	—
**Race/Ethnicity^§§^**								
White	21,843	34.2 (33.8–34.7)	2001–2014	0.3 (-0.3–0.9)	—	—	—	—
Black	3,129	18.7 (18.0–19.3)	2001–2014	1.2 (-0.1–2.7)	—	—	—	—
Hispanic	10,595	42.9 (42.1–43.7)	2001–2008	2.5 (0.3–4.7)**	2008–2014	-1.8 (-4.2–0.6)	—	—
American Indian/Alaska Native	350	30.2 (27.1–33.6)	2001–2014	-1.9 (-4.2–0.5)	—	—	—	—
Asian/Pacific Islander	1,765	31.6 (30.1–33.1)	2001–2014	0.3 (-0.9–1.6)	—	—	—	—
**U.S. Census region^¶¶^**								
Northeast	—***	34.8 (34.0–35.6)	2001–2007	3.0 (0.2–6.0)**	2007–2014	-1.6 (-3.7–0.7)	—	—
Midwest	—	32.4 (31.7–33.2)	2001–2011	1.6 (0.6–2.6)**	2011–2014	-5.4 (-11.4–1.0)	—	—
South	—	31.6 (31.0–32.1)	2001–2003	-4.6 (-15.3–7.6)	2003–2008	3.9 (0.2–7.7)**	2008–2014	-1.3 (-3.2–0.5)
West	—	38.5 (37.8–39.3)	2001–2014	0.4 (-0.3–1.1)	—	—	—	—
**County-level economic status by percentile (%)**								
Bottom 25	4,182	32.2 (31.2–33.2)	2001–2014	1.4 (0.6–2.2)**	—	—	—	—
25–75	22,141	33.9 (33.4–34.3)	2001–2010	1.1 (0.2–2.1)**	2010–2014	-2.4 (-5.5–0.9)	—	—
Top 25	10,646	34.9 (34.2–35.6)	2001–2008	2.9 (0.7–5.1)**	2008–2014	-1.5 (-4.0–1.1)	—	—
**Urban/Rural status**								
Metropolitan area ≥1 million population	21,690	35.7 (35.3–36.2)	2001–2008	2.7 (1.2–4.2)**	2008–2014	-1.6 (-3.4–0.2)	—	—
Metropolitan area 250,000 to <1 million population	8,134	34.4 (33.7–35.2)	2001–2011	0.8 (-0.4–2.1)	2011–2014	-4.4 (-11.8–3.7)	—	—
Metropolitan area <250,000 population	3,302	33.8 (32.7–35.0)	2001–2014	0.6 (-0.3–1.5)	—	—	—	—
Nonmetropolitan counties	4,962	32.9 (32.0–33.9)	2001–2014	0.9 (0.0–1.8)	—	—	—	—

**Source:** CDC’s National Program of Cancer Registries and the National Cancer Institute’s Surveillance, Epidemiology, and End Results program.

**Abbreviation**: CI = confidence interval.

* Per 1 million persons, age-adjusted to the 2000 U.S. standard population.

^†^ Cases included *International Classification of Diseases for Oncology, Third Edition* codes (9728–9729, 9811–9818, 9835–9837) as grouped by the *International Classification of Childhood Cancer*.

^§^ Incidence data are compiled from cancer registries that meet the data quality criteria for all years during 2001–2014 (covering approximately 98% of the U.S. population). Registry-specific data quality information is available at https://www.cdc.gov/cancer/npcr/uscs/data/00_data_quality.htm. Characteristic values with unknown, other, missing, or blank results are not included in this table.

^¶^ Trends were measured with APC in rates and were considered to increase or decrease if p<0.05; otherwise trends were considered stable. Trends were calculated using joinpoint regression, which allowed for different slopes in as many as three different periods, represented by APC_1_, APC_2_, and APC_3_, as applicable. The duration in years of APC_1_, APC_2_, and APC_3_ varied by study characteristic depending on joinpoint regression calculation. APC was not calculated if case count was <16 cases in any 1 year.

** p<0.05.

^††^ Trend adequately described during 2001**–**2014 by previous APC columns.

^§§^ White, black, American Indian/Alaska Native, and Asian/Pacific Islander persons are non-Hispanic. Hispanic persons might be of any race.

^¶¶^ *Midwest*: Illinois, Indiana, Iowa, Kansas, Michigan, Minnesota, Missouri, Nebraska, North Dakota, Ohio, South Dakota, and Wisconsin. *Northeast*: Connecticut, Maine, Massachusetts, New Hampshire, New Jersey, New York, Pennsylvania, Rhode Island, and Vermont. *South*: Alabama, Arkansas, Delaware, District of Columbia, Florida, Georgia, Kentucky, Louisiana, Maryland, Mississippi, North Carolina, Oklahoma, South Carolina, Tennessee, Texas, Virginia, and West Virginia. *West*: Alaska, Arizona, California, Colorado, Hawaii, Idaho, Montana, Nevada, New Mexico, Oregon, Utah, Washington, and Wyoming.

*** Number counts suppressed per United States Cancer Statistics complementary cell suppression rules: counts for national and regional data must be suppressed if a single state in a region or division is suppressed.

**FIGURE 1:**
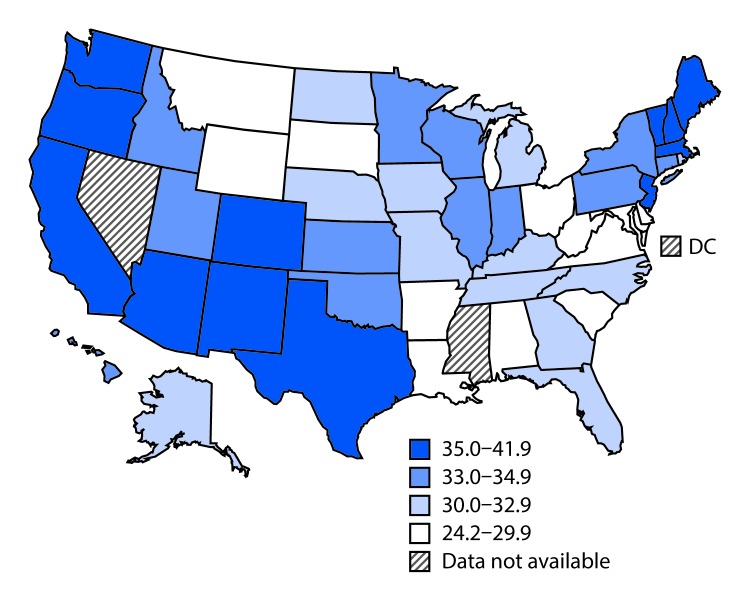
Annual age-adjusted rates* of acute lymphoblastic leukemia among persons aged <20 years, by state — National Program of Cancer Registries, and Surveillance, Epidemiology, and End Results program, United States, 2001–2014 * Rates are per 1 million persons and age-adjusted to the 2000 U.S. standard population.

Overall pediatric ALL incidence increased 1.9% per year during 2001–2008, and then remained stable during 2008–2014 (Figure 2). Incidence increased among males during 2001–2008 and among children aged 5–9 years and 10–14 years during 2001–2010 and 2001–2014, respectively, as well as in metropolitan areas with populations ≥1 million during 2001–2008 (Table). Among Hispanics, rates increased during 2001–2008 (APC 2.5, 95% confidence interval = 0.3–4.7) and were stable (nonsignificant decrease) during 2008–2014; pediatric ALL incidence rates were stable in all other racial/ethnic groups. State-specific analysis indicated that pediatric ALL incidence increased during all or part of 2001–2014 in four states: Alabama, Maryland, Massachusetts, and New York (Supplementary Table 1, https://stacks.cdc.gov/view/cdc/47662).

**FIGURE 2:**
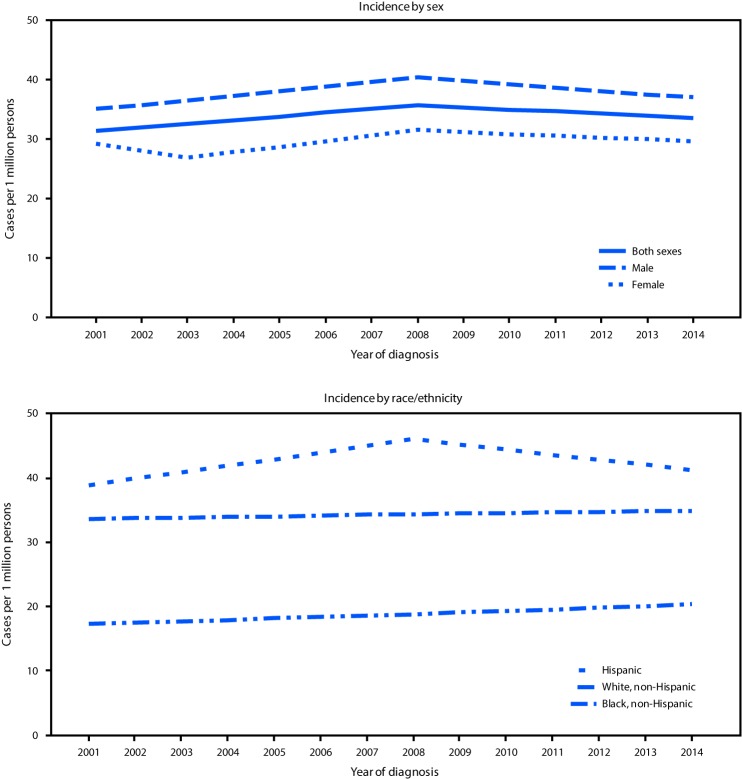
Trends* in age-adjusted rates^†^ of acute lymphoblastic leukemia in persons aged <20 years, by sex^§^ and race/ethnicity^¶^ — National Program of Cancer Registries, and Surveillance, Epidemiology, and End Results program, United States,** 2001–2014 * Trends were measured with annual percent change (APC) in rates, calculated using joinpoint regression, which allowed different slopes for as many as three different periods. ^†^ Rates are per 1 million persons and age-adjusted to the 2000 U.S. standard population. ^§^ APC for acute lymphoblastic leukemia for both sexes and for males was significantly different from zero during 2001–2008. ^¶^ APC for acute lymphoblastic leukemia for Hispanics was significantly different from zero during 2001–2008. ** Incidence data are compiled from cancer registries that meet the data quality criteria for all years 2001–2014 (covering approximately 98% of the U.S. population). https://www.cdc.gov/cancer/npcr/uscs/data/00_data_quality.htm.

## Discussion

Consistent with other published data, this analysis found that rates of ALL were highest in males, children aged 1–4 years, and Hispanics (*1*). Rates varied by state and region and were highest in the West U.S. Census Region. This report, using more recent data with broader population coverage than past studies (*1*–*3*), confirms an increase in pediatric ALL overall and among Hispanics (2001–2008) and also documents a subsequent period of stable trends overall and among Hispanics (2008–2014).

High rates of pediatric ALL in the Hispanic population might explain high ALL rates in the West U.S. Census Region and in other specific states, given the high proportion of Hispanics in many of these areas.^††^ Past studies documenting increasing incidence of pediatric ALL in Hispanics focused on earlier periods using the Surveillance, Epidemiology, and End Results database or the California Cancer Registry (*2*,*3*). Recent stable trends in ALL rates in Hispanic populations (2008–2014) might indicate a change after 2 decades of documented increasing trends. The cause for the higher rates of ALL in Hispanic populations and the increase during 2001–2008 is unknown; however, past studies have evaluated such factors as genetic susceptibility, disproportionate environmental exposure to household chemicals, or racial and ethnic disparities in parents’ exposures to chemicals at work (*2**,**3*). Other studies have hypothesized that the increasing trends in obesity among Hispanics might explain the increasing trends of ALL incidence among this population (*2*).

This report documents higher rates of ALL in persons aged <20 years living in counties in metropolitan areas with ≥1 million population and in counties in the top 25th income percentile. Past studies of pediatric leukemia have investigated possible associations with higher economic status or increased exposure to air pollution that is often found in large metropolitan areas (*5*,*6*). Etiologic studies examining potential causes of pediatric leukemia have documented associations between leukemia and exposures to solvents, traffic, pesticides, tobacco smoke, or radiation, or to specific nutritional exposures (*7*).

The findings in this report are subject to at least five limitations. First, the District of Columbia, Mississippi, and Nevada were excluded because of incomplete trend data, which limits the representativeness of the results. Second, although the United States Cancer Statistics data publication standards yield high quality data, misclassifications of race and ethnicity exist and might underestimate rates in American Indians, Alaska Natives, and Hispanics (*8*); ongoing procedures are used to ensure that this information is as accurate as possible. Third, the U.S. Census population estimates used in rate denominators might undercount some groups, including children and Hispanics, which could artificially raise incidence rates (*3*). Fourth, improvement in case ascertainment through advancements in electronic pathology reporting might affect trends: rates might appear to increase because current cancer registration methods are more accurately recording cases that were previously under-recorded. Finally, the possibility of a statistical error exists when analyzing subgroups with small numbers. Although APCs that are close to the significance cutoff might be truly significant, future studies will be needed to validate and monitor trends.

These recent state-based demographic cancer data can help local and national cancer control programs assess needs, allocate resources, and guide policy and public health strategies that can reduce cancer risk and improve the care of children and adolescents with ALL. Because cancer clinical trial participation has become an increasingly important part of quality clinical care (*9*), many state health departments have created cancer control plans that aim to address the economic and sociocultural barriers that limit certain groups enrolling in these trials.^§§^ Knowledge about rates and trends of pediatric ALL might help tailor the goals of these programs to address local and disease-specific needs. In addition, as incidence and survival of pediatric ALL increase (*1*), public health professionals can use recent ALL incidence data to improve local cancer survivorship programs that address chronic disease management, screen for late effects, and provide resources to help patients maintain a high quality of life (*10*). Public health planners can prioritize issues pertinent to pediatric cancer survivors such as transitioning to adult care and accessing the educational resources that might be available to these patients. Finally, health care professionals and researchers can use surveillance data to inform research questions. Continued surveillance data will be needed to further track incidence changes and public health needs relative to specific demographic groups and geographic areas.

**Summary**

**What is already known about this topic?**

Acute lymphoblastic leukemia (ALL) is the most common cancer in children and adolescents in the United States. Past studies using ≤28% population coverage have described increasing incidence of pediatric ALL, especially in Hispanic populations.

**What is added by this report?**

Analysis of data covering 98% of the U.S. population indicated that the incidence of pediatric ALL increased during 2001–2008 overall and for Hispanics, but then was stable during 2008–2014. The cause for the higher rates of ALL in Hispanic populations and the increase during 2001–2008 is unknown. Incidence of pediatric leukemia during 2001–2014 was highest in the West U.S. Census Region, possibly reflecting the high proportion of Hispanics in many of the region’s constituent states.

**What are the implications for public health practice?**

Increasing incidence of pediatric ALL in certain demographic groups might necessitate changes to cancer control planning, affecting treatment and survivorship care. Continued cancer surveillance will be important in guiding future research, including etiologic studies.

## Footnotes

* Cancer registries’ incidence data met the following five United States Cancer Statistics criteria: 1) ≤5% of cases ascertained solely on the basis of death certificate; 2) ≤3% of cases missing information on sex; 3) ≤3% of cases missing information on age; 4) ≤5% of cases missing information on race; and 5) ≥97% of registry’s records passed a set of single-field and inter-field computerized edits that test the validity and logic of data components. https://nccd.cdc.gov/uscs/.

† http://codes.iarc.fr/.

§ https://seer.cancer.gov/iccc/iccc-who2008.html. “ALL” in this study includes precursor cell leukemia and lymphoma *International Classification of Diseases for Oncology, Third Edition* codes, excluding code 9727 because of its use to code for blastic plasmacytoid dendritic cell neoplasm. Additional information is available at http://onlinelibrary.wiley.com/doi/10.1002/cncr.20910/full and http://www.bloodjournal.org/content/114/5/937?sso-checked=true.

¶ Population estimates incorporate bridged single-race estimates derived from the original multiple race categories in the 2010 U.S. Census. https://seer.cancer.gov/popdata.

** https://www.census.gov/geo/reference/gtc/gtc_census_divreg.html.

†† https://www.census.gov/prod/cen2010/briefs/c2010br-04.pdf.

§§ https://www.cdc.gov/cancer/ncccp/index.htm.
